# RETRACTION: The Hepatic and Pancreatic Tumour Resection Risk Factors for Surgical Site Wound Infections: A Meta‐Analysis

**DOI:** 10.1111/iwj.70201

**Published:** 2025-02-02

## Abstract

**RETRACTION:** DongB. , ChenJ. , SongM. , YouC. , LeiC. , and FanY. , “The Hepatic and Pancreatic Tumour Resection Risk Factors for Surgical Site Wound Infections: A Meta‐Analysis,” International Wound Journal 20, no. 8 (2023): 3140–3147, 10.1111/iwj.14190.37194335 PMC10502255

The above article, published online on 16 May 2023, in Wiley Online Library (http://onlinelibrary.wiley.com/), has been retracted by agreement between the journal Editor in Chief, Professor Keith Harding; and John Wiley & Sons Ltd. Following an investigation by the publisher, all parties have concluded that this article was accepted solely on the basis of a compromised peer review process. The editors have therefore decided to retract the article. The authors did not respond to our notice regarding the retraction.

**RETRACTION:** B. Dong , J. Chen , M. Song , C. You , C. Lei , and Y. Fan , “The Hepatic and Pancreatic Tumour Resection Risk Factors for Surgical Site Wound Infections: A Meta‐Analysis,” International Wound Journal 20, no. 8 (2023): 3140–3147, 10.1111/iwj.14190.37194335 PMC10502255

The above article, published online on 16 May 2023, in Wiley Online Library (http://onlinelibrary.wiley.com/), has been retracted by agreement between the journal Editor in Chief, Professor Keith Harding; and John Wiley & Sons Ltd. Following an investigation by the publisher, all parties have concluded that this article was accepted solely on the basis of a compromised peer review process. The editors have therefore decided to retract the article. The authors did not respond to our notice regarding the retraction.

